# Supplementary material for: “Our kids are our future”: Barriers and facilitators to vaccine uptake and timeliness among Aboriginal children younger than five years in Boorloo (Perth), Western Australia
Source: PLoS One. 2025 May 19;20(5):e0317946. doi: 10.1371/journal.pone.0317946 (PMC12087994; doi:10.1371/journal.pone.0317946)
Supplement: S1 File — (DOCX) [file pone.0317946.s001.docx]

Inclusivity in global research

PLOS’ policy on inclusivity in global research aims to improve transparency in the reporting of research performed outside of researchers’ own country or community and ensures that PLOS publications reporting global research adhere to high standards for research ethics and authorship. Authors of relevant research articles may be asked to complete the questionnaire below, which outlines ethical, cultural, and scientific considerations specific to inclusivity in global research. This questionnaire may be requested when researchers have travelled to a different country to conduct research, if research uses samples collected in another country, research with Indigenous populations or their lands, or if research is on cultural artefacts. Researchers travelling to another country solely to use laboratory equipment will not normally be required to complete the questionnaire. However, the questionnaire can be requested at the journal’s discretion for any submission – if you have been requested to complete this questionnaire by the PLOS journal you submitted to, please do so.

Please complete the questionnaire below and include this as a Supporting Information file with your manuscript. Note that if your paper is accepted for publication, this checklist will be published with your article in the supporting information files. Please ensure that you reference the checklist in the main body of your manuscript. We suggest adding a subsection ‘Inclusivity in global research’ to your Methods section and adding the following sentence: “Additional information regarding the ethical, cultural, and scientific considerations specific to inclusivity in global research is included in the Supporting Information (SX Checklist)”

The questions have been designed to be applicable to a wide range of study types, and there are subsections for both human subjects research and non-human subjects research. If any of the questions are not relevant to your research please mark them as “N/A” as appropriate.

**Ethical considerations, permits and authorship**

*This section is applicable to all research types.*

Provide details as to who granted permissions and/or consent for the study to take place in the Methods section of your manuscript. This should include the names of **all** ethics boards, governmental organizations, community leaders or other bodies that provided approval for the study. If individuals provided approval refer to these people by their role or title but do not list their name(s).

Reported on page number: 7

If there were any deviations from the study protocol after approval was obtained please provide details of these changes in the Methods section of your manuscript.
Did this study involve local collaborators that are residents of the country where the research was conducted or members of the community studied? If you do not have any authors from said communities, please provide an explanation for this below.

Reported on page number: N/A

Yes, this study involved six authors who identify as Aboriginal: PWK, NN, JH, JK, MR and VS.

Of notable mention, were the contributions made by PWK and VS. PWK took a leading role in community consultation, project design and administration, data analysis and the provision of ongoing cultural oversight. Overarching cultural governance and support was provided by VS who is a senior Aboriginal woman and employed as the Aboriginal Cultural Guidance Advisor within the Wesfarmers Centre of Vaccines and Infectious Diseases at the Kids Research Institute Australia. For a full list of contributions, please refer to ‘Author contributions’ in the manuscript.

Everyone listed as an author should meet PLOS’ criteria for authorship and all individuals who meet these criteria should be included in the author byline, rather than the acknowledgements. For further information please see the journal’s Authorship Policy.

**Human subjects research (e.g. health research, medical research, cross-cultural psychology)**

Did you obtain written informed consent from a representative of the local community or region before the research took place? How did you establish who speaks for the community? Details of written informed consent obtained from study participants should be reported separately in the Methods section of your manuscript.

There is no single Indigenous authority in our study region, who speaks for the community. However, as a condition of ethics approval, the WA Aboriginal Health Ethics Committee require researchers obtain a letter of support from the Kulunga Aboriginal Unit. This unit is endorsed by the WA Aboriginal Health Ethics Committee as having the authority to provide written informed consent to conduct research studies conducted at The Kids Research Institute Australia, involving Aboriginal and/or Torres Strait Islander peoples in the Perth metropiltan region. We received a letter of support from the Kulunga Aboriginal Unit for this research project on 13 May 2022.

Additionally, since its inception, there has been ongoing Aboriginal governance and oversight of the project to ensure the presence of Aboriginal voices throughout.

How did members of the local community provide input on the aims of the research investigation, its methodology, and its anticipated outcome(s)?

Throughout 2022, we hosted three pre-study workshops with Aboriginal Elders, parents/carers of Aboriginal and/or Torres Strait Islander children, and those working in Aboriginal health. These workshops sought to: 1) garner support for our project (and to learn if this was a topic that members of the Boorloo Aboriginal community wanted researched), 2) acquire feedback on our proposed methods, and 3) identify community members’ understanding of potential barriers to vaccination, thereby helping to develop the interview questions. Through these workshops, members of the local community were able to provide input into the study aims, study design, methodology and anticipated outcomes.

Additionally, prior to seeking human ethics approval to conduct this research, we also presented our proposed study to a panel of both Aboriginal and non-Aboriginal researchers and community members from across the country. Members of this panel were able to provide input and advice to reseachers on their projects, to ensure they are being caried out in a culturally safe and meaningful way.

When engaging with the local community, how did you ensure that the informed consent documents and other materials could be understood by local stakeholders?

Project documents including participant information forms, participant consent forms, demographic survey questions and interview questions were reviewed by two Aboriginal community members to ensure the language used was culturally and linguistically appropriate. These community members were provided with an honarium payment for their review of these documents. A condition of eligibility for this project was that participants could read and understand English. Prior to commencing data collection, researchers spent time describing the study to parents/carers in detail (which included thoroughly talking through each section of the participant information and consent forms). If parents/carers were interested in participating, researchers then obtained informed written consent to proceed.

Will the findings of the research be made available in an understandable format to stakeholders in the community where the study was conducted (e.g. via a presentation, summary report, copies of publications, etc.)? Please provide details of how this will be achieved.

The findings of the research were presented to stakeholders and community members at a Community Forum in April 2024. At this forum, the research team shared preliminary findings and sought verbal feedback on whether the findings resonated with attendees, and how the findings could be communicated

about in most respectful way. Final themes presented in this paper incorporate the feedback received.

Additionally, project findings were presented to the Our Children’s Health Research Aboriginal Advisors (OCHRAA) through the Wesfarmers Centre of Vaccine and Infectious Diseases on 30 July 2024. This group is made up of 15 Aboriginal community members from across Western Australia (not just Boorloo) and include Aboriginal Elders, health care workers, parents and grandparents. Advisors in this group are committed to sharing project findings through their networks to aid researchers in disseminting results widely.

Lastly, one-page plain language summary reports will be produced and disseminated to partner organisations to share with their clients and other members of the Boorloo Aboriginal community.

**Non-human subjects research using specimens/ animals collected as part of the study, or those housed in archival collections. Examples include archaeology, paleontology, botany and zoology.**

Did the permission you obtained from a local authority to perform the study include an agreement on access to outputs and benefit sharing? This may include procedures to enable fair distribution of the benefits and resources arising from the research performed. Please include any details of Prior Informed Consent and Benefit Sharing Agreements obtained. These may be required by field-specific regulations, for example the Convention on Biological Diversity (CBD) and the associated Nagoya Protocol.

N/A

If the material used in your study was imported, please A) provide the year it was imported and B) indicate whether permits were obtained to import/export the materials used, C) provide details of any permits obtained. If this information is not available, please indicate this.

N/A

If you used archival specimens, please state how the material used in your study was acquired by the institute it is held in and provide details of any permits obtained for the original excavations/ sample collection. If this information is not available, please indicate this.

N/A

How was the potential cultural significance of the materials collected in your study to local communities considered in your research design? Were Indigenous peoples and/or local researchers and institutions involved with archaeological excavations / collection of specimens? If so, please provide a description of their involvement.

N/A

If your manuscript includes photographs of human remains please indicate whether authors obtained permission from descendants or affiliated cultural communities to do so.

N/A
